# Gender differences in the factors predicting initial engagement at cardiac rehabilitation

**DOI:** 10.1136/openhrt-2017-000764

**Published:** 2018-03-27

**Authors:** Paul Michael Galdas, Alexander Stephen Harrison, Patrick Doherty

**Affiliations:** Department of Health Sciences, Faculty of Science, University of York, York, UK

**Keywords:** cardiac rehabilitation, prevention, gender, observational study

## Abstract

**Objective:**

To determine whether there are gender differences in the factors that predict attendance at the initial cardiac rehabilitation baseline assessment (CR engagement) after referral.

**Methods:**

Using data from the National Audit of Cardiac Rehabilitation, we analysed data on 95 638 patients referred to CR following a cardiovascular diagnosis/treatment between 2013 and 2016. Eighteen factors that have been shown in previous research to be important predictors of CR participation were investigated and grouped into four categories: sociodemographic factors, cardiac risk factors, patient medical status and service-level factors. Logistic binary regression models were built for male patients and female patients, assessing the likelihood for CR engagement. Each included predictors such as age, number of comorbidities and social deprivation score.

**Results:**

There were no important differences in the factors that predict the likelihood of CR engagement in men and women. Seven factors associated with a reduced probability of CR engagement, and eight factors associated with increased probability, were identified. Fourteen of the 15 factors identified as predicting the likelihood for engagement/non-engagement were the same for both men and women. Increasing age, being South Asian or non-white ethnicity (other than Black) and being single were all associated with a reduced likelihood of attending an initial CR baseline assessment in both men and women. Male patients with diabetes were 11% less likely to engage with CR; however, there was no significant association in women. Results showed that the overwhelmingly important determinant of CR engagement observed in both men and women was receiving an invitation to attend an assessment session (OR 4.223 men/4.033women; p<0.05).

**Conclusions:**

Consideration of gender differences in predictors of CR uptake should probably be more nuanced and informed by the stage of the patient care pathway.

**Key questions**

**What is already known about this subject?**

- Gender differences in the factors influencing cardiac rehabilitation (CR) participation are well established in the literature, and tailored gender-specific interventions to promote access have been recommended on this basis. National audit data of CR services suggest that gender-specific interventions are not yet part of routine practice and that 50% of eligible patients fail to take up any form of CR. Little data are available, from research in routine practice, on whether gender differences exist in the factors predicting attendance at the initial CR baseline assessment (CR engagement), which informs the design of a tailored CR programme.

**What does this study add?**

- This is the first UK-based study, using data that reflect routine practice, showing there are no important gender differences in the factors that predict attendance at the initial CR baseline assessment for patients with cardiovascular diagnosis or undergoing cardiac treatment following a heart attack. Increasing age, being South Asian or non-white ethnicity (other than Black) and being single were all associated with a reduced likelihood of attending the initial baseline assessment in both men and women. The overwhelmingly important determinant of CR engagement observed in both men and women was receiving an invitation to attend an assessment session.

**Key questions**

**How might this impact on clinical practice?**

- Early engagement with the CR pathway is vital for ensuring programme uptake and adherence and the achievement of meaningful clinical outcomes. Our findings provide a steer to health professionals on which patients are less likely to attend an initial baseline assessment so that strategies aiming to optimise CR engagement can be adapted and tailored. Results from the current study suggest that gender-specific strategies may not be appropriate at this early stage of the CR pathway. Efforts to enhance initial CR engagement should instead focus on ensuring all patients receive an invitation to attend an assessment, with particular attention paid to patients who are single, older, non-English speakers and from lower socioeconomic groups.

## Introduction

Attendance at and completion of cardiac rehabilitation (CR) programmes is poor worldwide. In the UK, recent analysis of National Audit of Cardiac Rehabilitation (NACR) data shows that uptake of CR among eligible patients is currently 50%.^1^ Although this places the UK in the top 2% of countries in Europe,^2^ uptake still remains below national recommendations of 65%–70%.^1^

**Figure 1:**
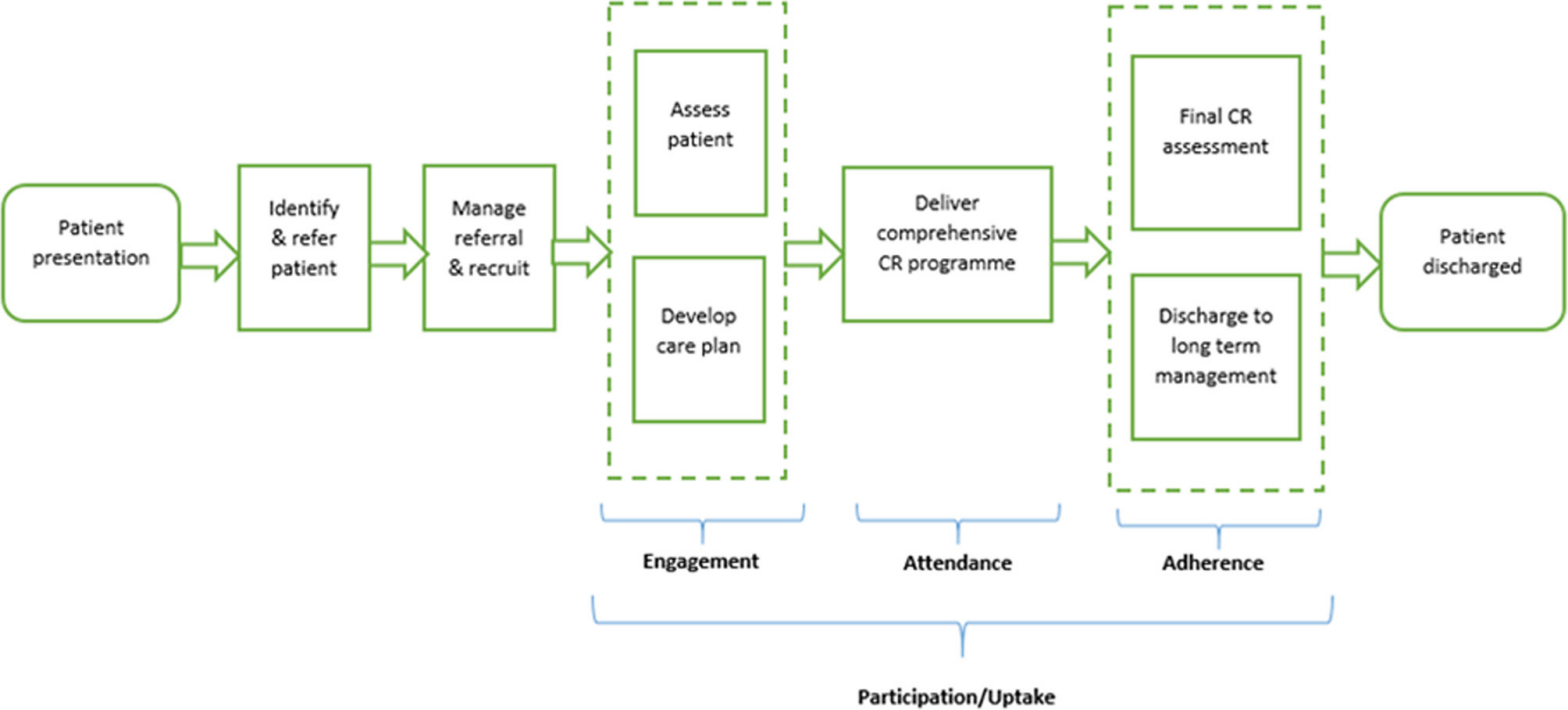
Department of Health commissioning guide six-stage patient pathway of care. CR, cardiac rehabilitation.

The CR pathway of care for patients following a cardiac event involves six stages (figure 1), each of which is vital for the achievement of meaningful clinical outcomes.^3^ Barriers to engagement, attendance and adherence within the pathway have been widely studied and shown to include patient-level factors (eg, illness perception, beliefs about treatment, social support, family responsibilities, work constraints); service-level factors (eg, programme accessibility, travel time, referral); sociodemographic factors (eg, older age, female, ethnic minority, low education levels, comorbidities); and psychological factors (eg, depression, anxiety).^4–9^

A range of strategies to increase the number of people participating in CR have been developed with these barriers in mind, such as motivational communications by nurse liaisons, therapists or peers; early appointments after discharge; gender-tailored CR; and intermediary rehabilitation programmes for older people.^10^ Although there is currently only weak evidence that these interventions are effective at improving participation, tailored approaches which aim to address social factors and patient-identified barriers have been recommended as the most likely to yield benefit.^6 10^

It is increasingly recognised that a ‘one-size-fits-all’ model will not be effective in the future,^11^ and that CR needs to be ‘rebranded and re-invigorated’ as a more tailored, person-centred intervention in order to reach a larger patient population.^12^ Gender-tailored CR interventions have been recommended as holding particular promise for improving uptake.^13 14^ A recurrent theme across qualitative studies of gender and CR experience is that women and men hold divergent views on their rehabilitation needs and their preferences on how exercise, group interaction and emotional support aspects of programmes are delivered.^15^ Gender has been shown to be a key variable in self-management decisions and preferences in a range of long-term conditions, including whether to attend CR-related support interventions.^16^ However, where gender-related barriers and solutions to CR attendance have been considered in the extant literature, women have tended to be the focus. Women’s lesser participation in CR programmes is widely recognised and has been extensively reviewed,^15 17–21^ with multilevel barriers including non-referral, lower education level, lack of social support and high burden of family responsibilities cited as key factors associated with poorer uptake.^13 17 18 20 22–26^

To date, research on the factors associated with men’s participation at CR has received little attention. Although men are more likely to take up CR than women, and be included in trials of CR effectiveness,^27^ male participation also remains suboptimal.^1^ More can be done to optimise uptake in both men and women if interventions to improve participation can be designed to address each group’s specific barriers. To this end, we undertook an analysis to determine whether there are gender differences in the patient-level and service-level factors that predict CR engagement, defined as attendance at the initial CR baseline assessment following referral. Our hypothesis was that the factors that predict men’s engagement with CR are different from those which are associated with women’s engagement.

## Methods

### Study design and population

We undertook a retrospective observational study using NACR data, a routinely collected clinical audit that collates information on patient characteristics, diagnosis/treatments and rehabilitation received. The audit collects information from over 300 CR programmes across England, Northern Ireland and Wales. Programmes entering data are approved by their Caldicott Guardian and the online system is hosted by NHS Digital. NHS Digital has approval to collect patient-identifiable data which are then anonymised and made available to the NACR team who validate the clinical quality of data entered. This data governance process removes the need for explicit consent from individual patients for the purposes of audit and service-related research under Section 251 of the NHS Act 2006 (https://www.legislation.gov.uk/ukpga/2006/41/pdfs/ukpga_20060041_en.pdf).

The current study used data from all patients with an initiating event (the primary diagnosis or treatment which resulted in patient becoming eligible for CR) between 1 April 2013 and 31 March 2016. Patients from conventional cardiovascular diagnosis/treatment groups (eg, coronary artery bypass graft (CABG), percutaneous coronary intervention (PCI) and medical management of myocardial infarction) were included. Heart failure diagnosis and its referral pathways are relatively new to the NACR with insufficient data capture at this point.

### Factors investigated

The current study examined patient ‘engagement’ within the CR pathway, defined as attendance at an initial assessment of individual patient needs following referral. We investigated 18 factors that have been shown in previous research to be important predictors of CR participation,^4–9 28^ illustrated in table 1. The inclusion of the sociodemographic variable, England Index of Multiple Deprivation (IMD), means that this study focused on the 186 programmes providing electronic data for CR in England.^1^ This variable is based on where patients reside and assigned at the Lower Socio Output Area. The service-level factors reflect all stages of the patient journey before reaching CR engagement. Table 1 shows the broad variables included in the analysis; subgroups such as the cardiac treatment, PCI, CABG and other are detailed in tables 2 and 3.

**Table 1:** Hypothesised predictors for CR engagement

Sociodemographic factors	Cardiac risk factors	Patient’s medical status	Service-level factors
Age	High blood pressure	Total number of comorbidities	Referred to CR
Ethnicity	Diabetes	Previous cardiac event	Venue of source of referral to CR
Marital status	High blood cholesterol	Angina	Hospital length of stay
Index of Multiple Deprivation	Anxiety		Received confirmed joining date
	Depression		Patient received early CR
	Family history		

CR, cardiac rehabilitation.

**Table 2:** Baseline characteristics of both groups

		Male			Female		
		Engaged	Not engaged	P values	Engaged	Not engaged	P values
n (%)		45 723 (66.5)	23 060 (33.5)		16 769 (62.4)	10 086 (37.6)	
Mean age (SD)		64 (12)	66 (13)	<0.001	68 (12)	71 (13)	<0.001
Ethnic group (White)	Black	0.90%	0.70%	<0.001	1.20%	0.70%	<0.001
	South Asian	7.30%	6.40%		5.60%	4.70%	
	Other	4.60%	6.10%		4.00%	5.30%	
Marital status (Single)	Partner	80.10%	76.80%	<0.001	62.60%	58.60%	<0.001
	Previous partner	10.70%	12.20%		29.20%	32.50%	
Comorbidity (No)	Hypertension	36.60%	30.50%	<0.001	39.70%	33.10%	<0.001
	Diabetes	17.00%	15.40%	<0.001	16.80%	15.10%	<0.001
	Hypercholesterolaemia/ dyslipidaemia	23.10%	19.90%	<0.001	21.40%	17.80%	<0.001
	Anxiety	3.60%	1.60%	<0.001	5.60%	2.10%	<0.001
	Depression. There shall be documented interaction between the patient and the multidisciplinary team.	3.80%	1.70%	<0.001	6.00%	2.40%	<0.001
	Lasting a minimum of 8 weeks						
	Family history	20.00%	13.00%	<0.001	20.70%	10.90%	<0.001
Number of comorbidities	<3	40.80%	34.10%	<0.001	37.80%	32.60%	<0.001
	3+	30.00%	22.70%		35.70%	26.10%	
Previous cardiac event study (No)	Yes	35.10%	33.90%	0.003	31.30%	30.30%	0.088
Comorbidity (No)	Angina	15.00%	11.20%	<0.001	14.50%	10.40%	<0.001
Mean length of stay (SD)		11 (28)	8 (15)	<0.001	11 (28)	9 (17)	<0.001
Invited to join date study (No)	Yes	78.20%	44.60%	<0.001	77.00%	43.90%	<0.001
Received early CR (No)	Yes	56.90%	66.10%	<0.001	57.70%	65.80%	<0.001
Referral setting (Hospital based)	Primary care setting	10.90%	9.20%	<0.001	10.50%	8.80%	<0.001
Cardiac treatment (No)	PCI	53.19%	47.06%	<0.001	47.13%	37.80%	<0.001
	CABG	16.37%	12.38%		8.99%	6.54%	
	Other	21.26%	23.71%		31.31%	33.14%	
Referral venue (NHS Trust)	General practice	3.30%	1.30%	<0.001	3.10%	1.20%	<0.001
	Private hospital	2.40%	2.20%		1.90%	1.90%	
Socioeconomic status (Lowest IMD quintile)	Second quintile	16.50%	19.90%	<0.001	17.80%	20.80%	<0.001
	Third quintile	19.80%	21.00%		19.70%	20.90%	
	Fourth quintile	22.70%	21.40%		22.30%	19.80%	
	Fifth quintile	26.60%	19.20%		24.10%	18.50%	

CABG, coronary artery bypass graft; CR, cardiac rehabilitation; IMD, Index of Multiple Deprivation; PCI, percutaneous coronary intervention.

**Table 3:** Pooled estimates of the logistic regression model predicting likelihood of CR engagement

Factor*	Categories	Male				Female			
		OR	95% CI for OR		P values	OR	95% CI for OR		P values
			Lower	Upper			Lower	Upper	
Age	Years	0.986	0.985	0.988	0	0.983	0.98	0.986	0
Ethnicity (White)	Black	1.282	1.032	1.594	0.025	1.278	0.936	1.746	0.123
	South Asian	0.89	0.825	0.959	0.002	0.834	0.73	0.953	0.008
	Other	0.685	0.631	0.743	0	0.694	0.603	0.797	0
Marital status (Single)	In partnership	1.332	1.25	1.42	0	1.329	1.19	1.485	0
	Previously partnered	1.295	1.192	1.407	0	1.47	1.302	1.66	0
IMD rank (1 most deprived)	2	1.1	1.032	1.172	0.003	1.138	1.032	1.254	0.009
	3	1.313	1.234	1.397	0	1.364	1.238	1.503	0
	4	1.408	1.324	1.498	0	1.57	1.425	1.73	0
	5	1.822	1.712	1.939	0	1.811	1.642	1.997	0
Cardiac risk factors (No)	Diabetes	0.89	0.84	0.943	0	Non-significant			
	Anxiety	1.343	1.164	1.549	0	1.463	1.215	1.763	0
	Depression	1.356	1.182	1.556	0	1.421	1.19	1.696	0
	Hypercholesterolaemia/ dyslipidaemia	0.779	0.738	0.822	0	0.785	0.721	0.854	0
	Family history	1.169	1.105	1.237	0	1.603	1.464	1.755	0
	Angina	1.142	1.073	1.217	0	1.221	1.104	1.352	0
Number of comorbidities (<3)	1–3	1.931	1.838	2.029	0	1.932	1.79	2.086	0
	>3	2.233	2.07	2.409	0	2.063	1.865	2.281	0
History of cardiac event (Yes)	No previous event	0.832	0.796	0.869	0	0.784	0.73	0.841	0
Hospital length of stay	Days	1.006	1.005	1.007	0	1.004	1.002	1.005	0
Received confirmed joining date (No)	Yes	4.223	4.06	4.393	0	4.033	3.789	4.294	0
Patient received early CR (No)	Yes	1.259	1.175	1.348	0	1.241	1.188	1.296	0
Cardiac treatment (No)	PCI	1.633	1.535	1.738	0	1.679	1.535	1.837	0
	CABG	1.789	1.659	1.929	0	1.797	1.575	2.052	0
	Other	1.313	1.226	1.406	0	1.335	1.216	1.466	0

The male and female models were statistically significant: X^2^ (25)=9818.053, p<0.001 and X^2^ (24)=4053.338, p<0.001. The models estimated R^2^ as 21.2%–22.3% (Nagelkerke R^2^) and the variance correctly classified 71.7%–72.6% of the cases.

* Factors included in table were included during backwards stepwise regression and met the inclusion threshold of p<0.05

CABG, coronary artery bypass graft; CR, cardiac rehabilitation; IMD, Index of Multiple Deprivation; PCI, percutaneous coronary intervention.

### Data analysis

Descriptive statistics were calculated to compare differences in baseline characteristics between engaged and non-engaged patients. The analysis was split by gender to assess the variation in demographics and the odds of engagement. The preliminary analysis used t-tests and χ^2^ tests for continuous and binary descriptive predictors.

Binary logistic regression models were built for each gender, which included predictors such as age, number of comorbidities and social deprivation score. The regression model tested the patient’s characteristics and pathway up to engagement with the likelihood of being assessed prerehabilitation (dependent variable). The model was a four-step backwards regression which inserted variables from sociodemographic factors through to service-level factors. This was the preferred model to assess the likelihood of patient engagement rather than differences which would have used a hierarchical design. The non-significant predictors were excluded based on p>0.05. The models were tested for log likelihood, variance and predictive power. The predictive power of the model was assessed through a receiver operator curve and the resulting area under the curve demonstrated its power. Interactions were tested within the model based on strong relationships between predictors.

To account for missingness within the data, all patients who had valid age, gender and marital status and had missing values >5% were subjected to multiple imputation. There were 20 iterations and the analysis displayed is the pooled versions. This was conducted using Rubin’s rule, the software SPSS V.24, and was conducted under the assumption that missing values are random.

## Results

**Figure 2:**
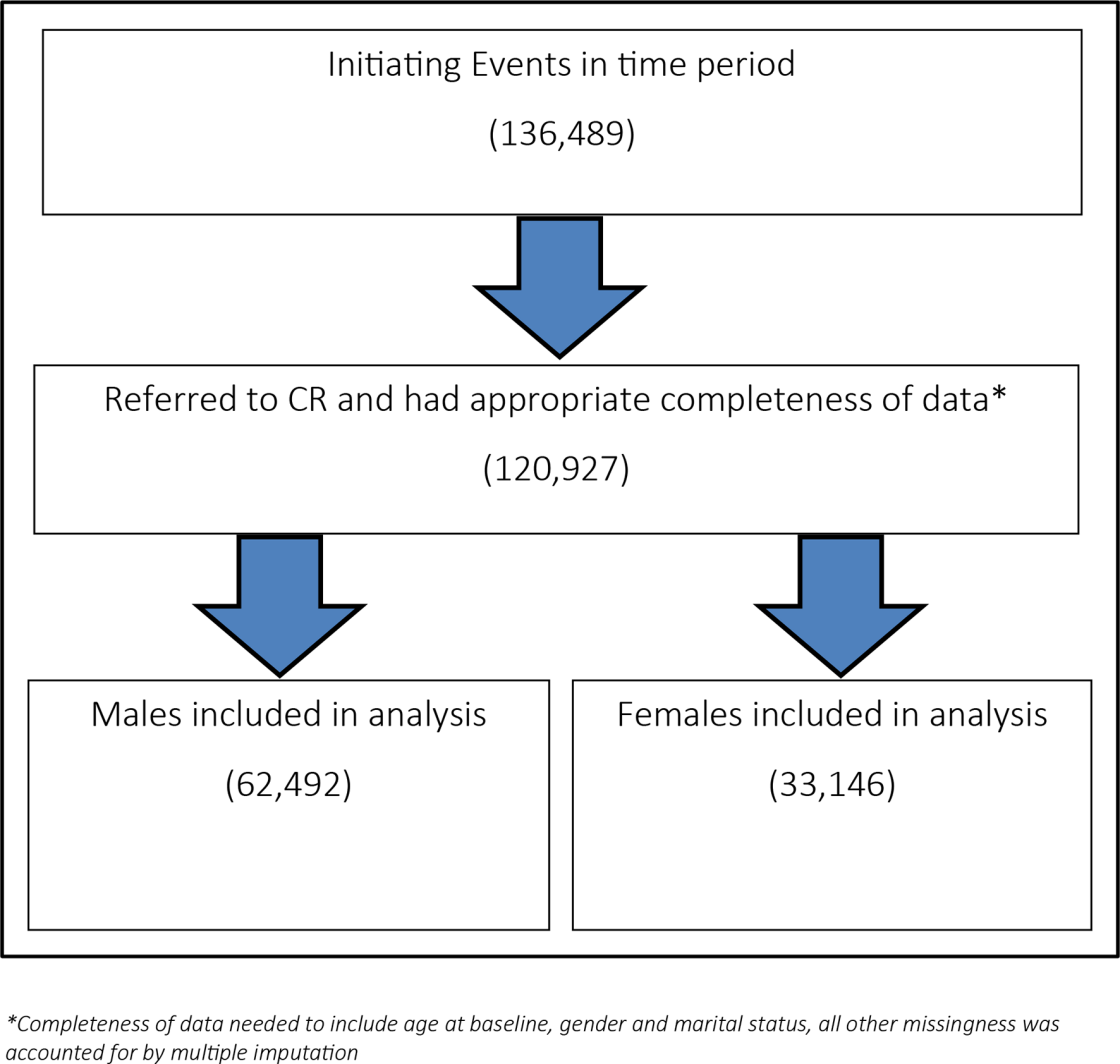
Flow diagram of the patients within study time period. CR, cardiac rehabilitation.

The analysis sample included 95 638 patients. The baseline characteristics of male and female participants (both engaged and not engaged in CR) are presented in table 2. Figure 2 presents a flow diagram of the patients within study time period (those who were referred to CR with an acceptable completeness of data) and the gender split of included patients.

Table 2 shows the differences between those patients receiving an initial assessment and those who did not. The total number of patients included in the regression analysis was 81 938 (59 232 male and 22 706 female); 13 700 were excluded. The regression model population included 45 086 patients who were processed using multiple imputation to account for missing values. The final models for each gender are shown in the online supplementary material.

10.1136/openhrt-2017-000764.supp1 Supplementary file 1

Table 3 presents the results from the regression. This shows that for all included predictors, the relationship between predictors and likelihood for engagement is the same for both men and women. The results from the regression presented an OR of 0.983–0.986 for 1-year increase in age. If we consider a 10-year increase this effect becomes 14%–17% less likely to attend, so a patient who is 65 years old in comparison to a 55-year-old patient, accounting for other factors, is 14%–17% less likely to achieve engagement.

If patients are from a higher socioeconomic area, as presented as quintile IMD, they are increasingly more likely to initially engage with CR. If patients identify as non-single, they are 29%–47% more likely to attend engagement than those who are single (men—OR partner 1.332 and previous partner 1.295, p<0.05; women—OR partner 1.329 and previous partner 1.470, p<0.05). There were no significant interactions between the factors included in the regression for either gender.

## Discussion

Gender differences in cardiovascular disease incidence, treatment, risk factor management and rehabilitation are observed worldwide.^29^ The need for better understanding and response to these differences is widely acknowledged.^30^ To our knowledge, the current study is the first quantitative investigation into gender differences in patient-level and service-level determinants of initial engagement with CR after referral (attendance at an initial baseline assessment). Our analysis has shown that there are no important differences in the factors that predict men’s and women’s likelihood of initial engagement with CR.

Early engagement with the CR pathway is vital for ensuring programme uptake and adherence and the achievement of meaningful clinical outcomes.^1 3^ Attendance at an initial assessment enables personalised goals to be identified and a tailored care plan to be agreed that meets individual needs, participation preferences and choices.^1 3^ Our findings provide a steer to health professionals on which patients are less likely to attend an initial assessment so that strategies aiming to optimise engagement can be adapted and tailored.^31^ The baseline characteristics of our nationally representative cohort of patients showed engagement with CR to be suboptimal in both men (66.5%) and women (62.4%). Regression analysis identified seven factors associated with a reduced probability of engagement, and eight factors associated with increased probability.

Consistent with the wider literature on factors associated with CR participation,^4–9 32^ increasing age, being South Asian or non-white ethnicity (other than Black) and being single were all associated with a reduced likelihood of attending an initial assessment in both men and women. Age was coded as continuous and showed a 10-year increase in age resulted in 14%–17% reduced likelihood of engagement in both genders.

Other factors identified as significant predictors of non-engagement in both men and women included having hypercholesterolaemia/dyslipidaemia (OR 0.78 men/0.79 women) and no history of a cardiac event (OR 0.83 men/0.78 women). If patients were from a lower socioeconomic area, as presented as quintile IMD, they were also increasingly less likely to engage with CR, with each group from 10%–13.8% going from 1 to 2 and 81%–82% from 1 to 5 (p<0.05).

Factors including having a partner, referral by general practitioner and having a PCI, CABG or other treatment were all predictive of attendance at initial assessment in both men and women. We identified only one factor where a significant difference between men and women was observed. Having diabetes was associated with an 11% reduced likelihood of engagement in men; an association that was not identified in women and the factor was removed from the final model. Previous qualitative research has found that men and women tend to report similar reasons for initial non-participation in CR^32^ and our findings corroborate this. Other than diabetes, it was striking that all factors identified as predicting the likelihood for engagement/non-engagement in the current study were the same for both men and women.

Aligned with the extensive literature on non-participation,^4–9 28^ the overwhelmingly important determinant of engagement that was observed in both men and women in the current study was receiving an invitation to attend an assessment session (OR 4.223 men/4.033 women; p<0.05). This suggests that efforts to enhance initial engagement should primarily focus on ensuring all patients receive an invitation to attend an assessment, with particular attention paid to patients who are single, older, non-English speakers and from lower socioeconomic groups.

Gender differences in the factors influencing attendance at and adherence to CR are well established in the literature,^6^ and targeted and tailored interventions to promote access have been recommended on this basis.^15 17–19 33^ We therefore hypothesised that there would be gender differences in the factors predicting initial engagement with CR after referral. The unexpected finding that there are no important gender differences at this important initial stage of the rehabilitation pathway suggests that a more nuanced view of how gender interplays with CR participation is required.

The decision to attend CR is multifactorial and intertwined with social contexts that afford inequitable opportunities for access.^15^ The current study adds weight to findings from recent systematic reviews and meta-analyses which indicate gender by itself may not always be the main determinant of CR uptake,^34^ and differences between men and women may diverge over the period of rehabilitation.^35^ This suggests that gender-specific strategies aiming to improve participation may be appropriate at some stages of the patient care pathway (eg, improving adherence) but not all (eg, promoting initial engagement). Further research into gender differences in determinants of CR attendance and adherence is warranted to fully determine this.

### Strengths and limitations of this study

The clinical relevance for identifying reasons for patients not attending initial assessment is significant. Early engagement is one of the British Association for Cardiovascular Prevention and Rehabilitation cornerstone core components^3^ and is the point in which patients’ CR is tailored and individual goals are agreed going forward. In investigating this stage of the pathway, we have identified key predictors which inhibit the progression of patients at an important stage of their CR journey. Future research should follow this cohort of patients along the CR pathway and investigate predictors at different stages, such as commencing and adhering to comprehensive programmes.

The regression was of good design with 21-2% R^2^ and 71-2% of cases being correctly classified. In this analysis, we included a multiple imputation technique which helped fill in missing data, and the population used was representative of modern CR patients in the UK. However, each year the completeness of data improves with the NACR, perhaps when coverage reaches a higher level in some years a redo of the analysis may confirm that the missingness was not a selection or reporting bias, although the authors are confident it is not and the multiple imputation was for increasing statistical power.

We found no important differences between men and women in 18 factors shown in previous research to be key predictors of CR participation.^4–9 28^ Although unlikely, it is possible that additional factors by which engagement does differ between genders were not included in our analysis.

Finally, including the IMD variable for a measure of social deprivation reduced the population to only England. Although the populations across the nations remain very similar each year, it is becoming more evident that the intercountry variations in terms of the CR offer and the structure within centres is diverse. The average wait times, length of core rehab and staffing profiles differ across countries, and in future research when a multicountry measure of social deprivation is available the authors would like to include Wales and Northern Ireland in the analysis.

## Conclusions

This is the first study into gender differences in the predictors of CR engagement, providing new insights into the factors that lead men and women to attend their initial CR baseline assessment (CR engagement) in a nationally representative cohort of patients. The key findings from the study have shown that there are no important differences in the factors that predict men’s and women’s likelihood of initial engagement with CR. Consideration of gender differences in predictors of CR uptake should probably be more nuanced and informed by the stage of the patient care pathway.
