# Supplementary material for: Gender differences in the factors predicting initial engagement at cardiac rehabilitation
Source: Open Heart. 2018 Mar 27;5(1):e000764. doi: 10.1136/openhrt-2017-000764 (PMC5888444; doi:10.1136/openhrt-2017-000764)
Supplement: Supplementary file 1 [file openhrt-2017-000764supp001.docx]

| Summary statistics for the four models created by forward stepwise regression. | | | | |
| --- | --- | --- | --- | --- |
|  | Model | -2 Log likelihood ratio | Pseudo-*R*_2_ | Correctly classified cases |
| Male | Model 1^a^ | 65145.4 | 0.03 | 67.30% |
|  | Model 2^b^ | 64411.14 | 0.05 | 37.50% |
|  | Model 3^c^ | 63626.68 | 0.07 | 67.60% |
|  | Model 4^d^ (final) | 57271.93 | 0.22 | 73.40% |
|  | Model | -2 Log likelihood ratio | Pseudo-*R*_2_ | Correctly classified cases |
| Female | Model 1^a^ | 25629.43 | 0.03 | 64.30% |
|  | Model 2^b^ | 25143.09 | 0.07 | 64.50% |
|  | Model 3^c^ | 24840.86 | 0.08 | 65.50% |
|  | Model 4^d^ (final) | 21521.62 | 0.21 | 71.89% |
| ^a^Socio-demographic factors only.  ^b^Model 1 plus risk factors.  ^c^Model 2 plus patient’s medical status.  ^d^Model 3 plus service level factors. | | | | |
